# Study of Spatio-Temporal Evolution, Integrated Prevention, and Control Measures of COVID-19 in the Yangtze River Delta

**DOI:** 10.1017/dmp.2022.297

**Published:** 2022-12-27

**Authors:** Li Yang, Haiyang Ren

**Affiliations:** 1 School of Management, Nanjing University of Posts and Telecommunications, Nanjing, China; 2 Beijing Institute of Surveying and Mapping, Beijing, China

**Keywords:** Yangtze River Delta, spatial analysis, comprehensive prevention and control measures

## Abstract

**Objective:**

The study analyzes the spatial characteristics of the epidemic. It evaluates the effectiveness of its differentiated prevention and control policies implemented at different stages of the epidemic in the Yangtze River Delta.

**Methods:**

The study divided the epidemic into 2 stages and analyzed the spatial evolution characteristics of the COVID-19 epidemic in the region by using Anselin Local Moran’s I and standard deviation ellipse.

**Results:**

In the first stage, the high value of confirmed cases was concentrated in the eastern and southern cities. The trajectory of the barycenter showed a V-shaped change characterized by a southward shift followed by a northward fluctuation. In contrast, the second stage was mainly concentrated in Jiangsu Province and Shanghai, and the Barycenter did not change over time. The diversified prevention and control measures enabled ‘zero new cases’ in the Yangtze River Delta within a month.

**Conclusion:**

The prevention and control policy implemented in the Yangtze River Delta has worked well. With the global pandemic of COVID-19, it is recommended that other countries follow the example of the Yangtze River Delta, tighten prevention policies, and speed up vaccination to avoid a rebound of the epidemic.

## Introduction

The global COVID-19 epidemic is currently at a high level^1^, and many countries must continue to rely on personal health measures to control the disease2 while promoting COVID-19 vaccination.^2^ However, vaccination and control measures are currently not being successfully implemented. The World Health Organization estimates that 500000 more people will die from COVID-19 in Europe by March, 2022 if no action is taken.^3^ In contrast, China has now entered a normalized phase of epidemic prevention and control at home, with nearly 2.4 billion doses of vaccination, but the continued risk of epidemic importation from abroad, combined with fall and winter influenza and other respiratory infectious disease epidemics, has greatly increased the complexity, and difficulty of epidemic prevention and control at home.^4^

The Yangtze River Delta is 1 of China’s most economically active regions. After the 2020 COVID-19 epidemic, the Yangtze River Delta region’s economy rebounded rapidly, surpassing the pre-epidemic level. Multiple outbreaks have occurred in the Yangtze River Delta between December 2019 and November 2021. The most recent outbreak centered at Lukou Airport in Nanjing, whose main strain is the Delta strain which has affected several provinces and cities both within and outside the Yangtze River Delta. Because the epidemic’s spread and transmission speed varied at different times in the Yangtze River Delta, the region implemented various prevention and control policies at various stages of the outbreak, ranging from city closures to small-scale control, and from vaccination to vaccine booster shots.

This study uses spatial analysis to examine the epidemic’s geographical distribution in the Yangtze River Delta from January 2020 to November 2021, and the epidemic policies undertaken in the region during various stages of the epidemic.

## Methods

The basic geographic information data were the base map of our spatial analysis in ArcGIS software (Redlands, California, USA). The city-level administrative division boundary containing the Yangtze River Delta was preprocessed using the WGS-84 coordinate system and obtained via National Geomatics Center of China.

This study used news from Lilac Garden, Sina News, and Tencent News to analyze policies and problems in the Yangtze River Delta.

Epidemic statistical data were used for spatial analysis. The epidemic data of Dingxiangyuan was gathered using the python crawling strategy and compared to data from Harvard University’s China Data Lab.^5^

Based on the characteristics of the epidemic in the Yangtze River Delta, the study divided the outbreak into 2 phases. The first phase was from January 22, 2020, to March 31, 2020, which was the outbreak phase when the COVID-19 epidemic was detected, and the second phase was from July 1, 2021, to October 1, 2021, which was the outbreak phase of the delta strain in the Yangtze River Delta. These 2 phases are the 2 periods of virus transmission in the Yangtze River Delta with many infections. There were no more significant outbreaks in the Yangtze River Delta during the other periods; therefore, only the outbreaks during these 2 time periods were considered in this study.

We collected epidemic statistics from January 10, 2020, to November 1, 2021. These statistics include the number of new and death cases daily in 2 stages. Our purpose was to analyze the data’s clustering characteristics to reflect the epidemic’s clustering characteristics.

Spatial autocorrelation is a spatial analysis method that can be used to analyze the spatial clustering characteristics of epidemics in a certain region. It can be divided into global autocorrelation and local autocorrelation.^6,7^ Global autocorrelation also includes local spatial positive correlation and local spatial negative correlation.^8^ This study used Anselin Local Moran’s I to indicate spatial variation characteristics of COVID-19 in the Yangtze River Delta. The main parameters of Anselin Local Moran’s I include conceptual spatial model, measurement construct, and standardization method.

Inverse distances are best for continuous data: the closer 2 features are in space, the more likely they will influence each other. Therefore, we chose the inverse distance as a conceptual spatial model. Euclidean and Manhattan are generally used when measuring spatial distance, but Manhattan is more suitable when the dataset has discrete or binary properties. Therefore we chose Euclidean as a measurement construct. Epidemic statistics just reflect the spatial characteristics of the epidemic. Hence, no standardized method was required.

Anselin Local Moran’s I can output the LISA map. The LISA map reflects the local indicators of spatial association (Local indicators of spatial association, abbreviated as LISA). In the LISA diagram, agglomeration is divided into 4 cases(‘high-high,’ ‘high-low,’ ‘low-high,’ and ‘low-low’), each of which identifies a region and its relationship to its neighbors (e.g., ‘high-low’ means that the center of the area is a high-value cluster, and the surrounding area is a low-value area).

In the meantime, to understand the direction of the central shift of COVID-19, we introduced the standard deviation ellipse (SDE), which can be used to identify the spatial directional characteristics and spread trend of the epidemic and discuss whether there are spatial shift characteristics.^9^ Usually, SDE has 2 main parameters: weight and ellipse size. SDE also has 3 levels of ellipses indicating that the generated ellipse can contain 68%, 95%, and 99% of data at 3 levels. We wanted to consider the main areas where the outbreak occurred, so the largest SDE was required, and the weights were confirmed cases and deaths.

## Results

### Spatial distribution characteristics of COVID-19 in the Yangtze River Delta

**Figure 1.**
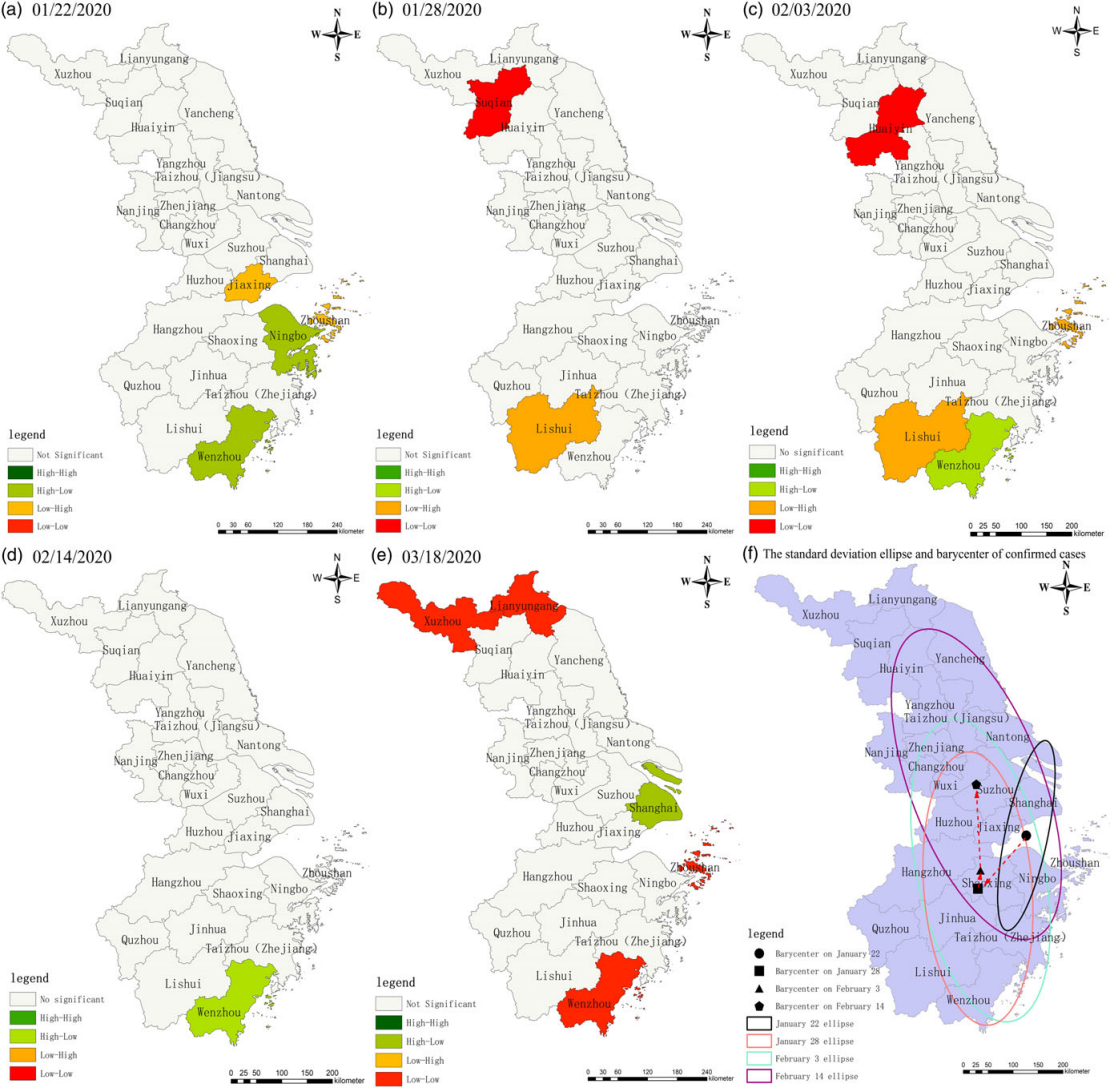
LISA plot of confirmed cases in the Yangtze River Delta (The first outbreak).

The study found that in the first outbreak (Figure 1), confirmed cases in the Yangtze River Delta had spatial clustering distribution characteristics with significant local autocorrelation. Clusters of high confirmed cases were primarily distributed in the eastern and southern cities, like Shanghai and Wenzhou, and clusters of low confirmed cases in the northern cities, like Xuzhou and Lianyungang. Spatial analysis showed that the cities with high risk were all related to imported cases in Hubei province in the first round. The cities with high concentrations of confirmed cases have good railway connections to Hubei Province.

**Figure 2.**
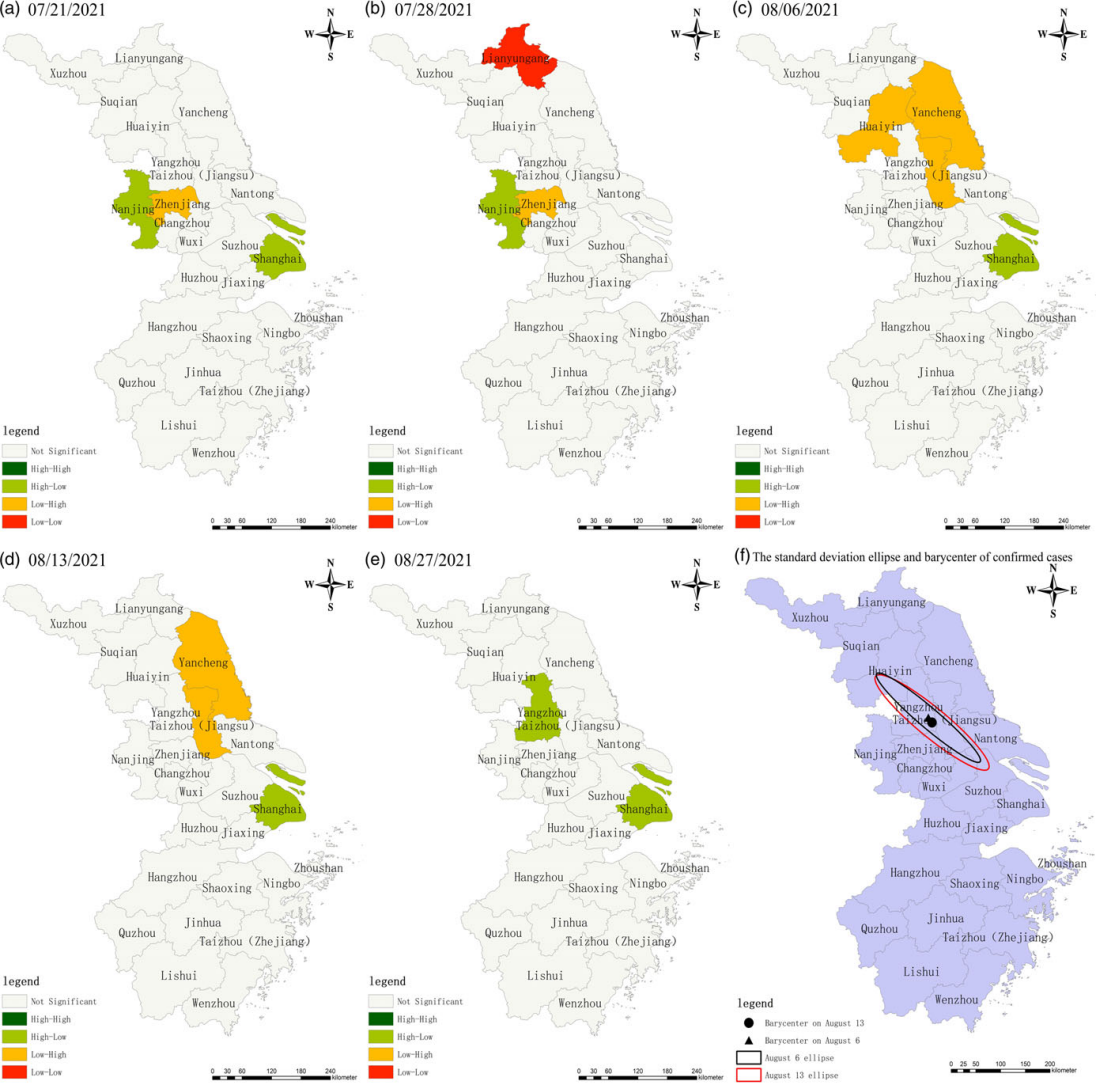
LISA plot of confirmed cases in the Yangtze River Delta (The second outbreak).

Figure 2 shows that the regional variance of confirmed cases in the second epidemic at different dates is significant. The spatial clustering area is primarily in Jiangsu Province and Shanghai, indicating significant local autocorrelation. On July 28, Nanjing reported 47 additional confirmed cases, making it the first epicenter of the epidemic, while Zhenjiang reported no new cases, indicating a ‘high-low’ and ‘low-high’ distribution, respectively. The low-value area is in Lianyungang City; 348 new cases were reported in Yangzhou City between August 6 and August 13, making it the epidemic’s second epicenter after Nanjing. Other cities in the Yangtze River Delta had no new confirmed cases on August 27, and Yangzhou and Shanghai had a few new cases, making them the ‘high-low’ gathering regions. Spatial analysis shows that the outbreak had mainly affected 3 cities, Nanjing, Yangzhou, and Shanghai. The cases in the Yangtze River Delta region were all related to imported cases, and the main transmission chain involved Lukou Airport.

### Gravity shift feature of COVID-19 in the Yangtze River Delta

The study created the standard deviation ellipse with the ellipse center, reflecting the epidemic’s spatial distribution’s center of gravity.^10^ The trajectory of the barycenter of confirmed cases in the first round of the epidemic showed a V-shaped characteristic of moving southward and then northward, and the spatial distribution of the number of cases tended to move southward and westward, according to the trend of the ellipse (Figure 1f). The confirmed case distribution shows an evident regional clustering phenomenon with a ‘northwest-southeast’ spatial distribution pattern. In general, confirmed cases were more concentrated in the Yangtze River Delta region’s center and southern cities, whereas northern cities fared better; most confirmed cases were imported from outside the municipality, with few intra-city outbreaks and inter-city transfers. Most of the strains involved in this cycle of epidemics are the original strain.

In the second stage, the barycenter of confirmed cases mainly remained constant (Figure 2f). The confirmed cases were grouped in Jiangsu and Shanghai, with no cases in Zhejiang Province, indicating a ‘northwest-southeast’ spatial distribution pattern. The number of confirmed cases in the Yangtze River Delta is relatively concentrated, with the epidemic’s epicenter in Nanjing in July and Yangzhou in August, while Zhejiang Province had virtually no cases. The majority of confirmed cases and associated cases are imported from other countries. The Delta strain makes up most cases in this cycle of outbreaks.

## Discussion

According to the characteristics of the epidemic in the Yangtze River Delta, we divided the COVID epidemic into 2 stages.

First, we used Anselin Local Moran’s I to analyze the aggregation of confirmed cases in the 2 stages. Clusters of high confirmed cases are primarily distributed in the eastern and southern cities, like Shanghai and Wenzhou, with clusters of low confirmed cases in the northern cities, like Xuzhou and Lianyungang. In the second stage, spatial analysis shows that the outbreak had mainly affected 3 cities: Nanjing, Yangzhou, and Shanghai.

This study showed that places with the highest risk were those with high population inflow from Hubei Province in the first outbreak. Similar studies show results consistent with this observation. A study conducted by Lei *et al*. showed that the places with the highest risk are those with high population inflow from Wuhan and Hubei Province.^11^ Majority of the confirmed and related cases in the second epidemic were imported from outside China, and the epidemic strain was Delta. The National Health Commission of China has proved that.^12^ The spatial clustering area is primarily in Jiangsu Province and Shanghai.

Second, we used the SDE to analyze the distribution direction of confirmed cases at different times to infer the transfer direction of the epidemic center. The trajectory of the barycenter of confirmed cases in the first stage showed a V-shaped characteristic of moving southward and then northward. In the second stage, the barycenter of confirmed cases mainly remained constant.

Finally, the epidemic prevention and control policies in the Yangtze River Delta are as follows. In the first outbreak, the Yangtze River Delta had established a joint prevention and control mechanism among cities during the epidemic.^13^ Lockdown and keeping social distance were common measures. In the second outbreak, the policies were different. Immunization had been hastened, and vaccine booster injections had been allowed. Small-scale controls, such as street or neighborhood closures and quarantines, will be implemented according to risk levels to stop the disease from spreading. For epidemic areas, a differentiated nucleic acid testing policy was introduced, with 3-day testing for epidemic areas inside the city until the requirement of no new cases for 21 days was met, and weekly testing for non-epidemic areas within the city, with a minimum of 3 tests. These policies brought the outbreak under control within months.

In this study, we compared the different policies applied to the 2 stages of the epidemic in the Yangtze River Delta. Our comparison may provide new thinking on epidemic prevention and control for other countries or regions.

## Conclusion

The study found that the combined prevention, control, and 0 transmission policies established in the Yangtze River Delta region were effective, based on the spatial distribution features and prevention and control policies of the 2 rounds of epidemic in the Yangtze River Delta. Given the current global crisis involving the COVID-19 pandemic, we recommend that other countries follow the example of the Yangtze River Delta region and implement the zero-COVID policy.
